# Nutritional status in irritable bowel syndrome: A North American population‐based study

**DOI:** 10.1002/jgh3.12311

**Published:** 2020-02-12

**Authors:** Isabel A Hujoel

**Affiliations:** ^1^ Division of Gastroenterology and Hepatology Mayo Clinic Rochester Minnesota USA

**Keywords:** brain–gut axis, copper–zinc ratio, dietary restriction, irritable bowel syndrome

## Abstract

**Background and Aim:**

Irritable bowel syndrome (IBS) affects 12% of the population, and the evidence supporting current medical interventions is poor. There is increasing focus on the therapeutic benefit of diet and supplementation. We aim to compare dietary composition and hematologic and biochemical markers in those with and without IBS to determine potential targets for therapeutic supplementation.

**Methods:**

All 17 national surveys between 1959 and 2019 were screened, and only 1, the Second National Health and Nutrition Examination Survey (NHANES II) (1976–1980), provided comprehensive data on IBS. We performed a cross‐sectional analysis of hematologic and biochemical markers and dietary composition of 12 295 individuals, aged 18–74, in NHANES II.

**Results:**

Individuals with IBS had significantly higher copper–zinc ratios (1.70 *vs* 1.55, *P* = 0.048) and were more likely to have ratios above 1.8 (odds ratio 1.79, 95% confidence interval 1.02–3.13), indicative of underlying copper–zinc imbalance. While more likely to report dietary avoidances, they had no other evidence of nutritional deficiencies. In addition, dietary recall showed that those with IBS consumed more calories (*P* = 0.02), were more likely to take vitamin supplements (*P* = 0.003), and that their macro and micronutrient intake was not significantly different.

**Conclusions:**

The findings suggest that individuals with IBS should be screened for copper–zinc imbalance. Given zinc's role in the immune system, the “brain–gut” axis, and the gastrointestinal barrier, the identified copper–zinc imbalance may play a role in perpetuating the underlying pathophysiology of IBS. Further studies are needed to investigate this hypothesis and the potential role of therapeutic zinc supplementation.

## Graphical abstract

Although individuals with irritable bowel syndrome (IBS) reported significantly more dietary avoidances, this study found that their consumption of micro‐ and macronutrients was not significantly different from those without IBS and that they in fact ate more calories. Hematologic and biochemical analysis did find that individuals with IBS were more likely to have elevated copper–zinc ratios. Further studies are needed to determine if this copper–zinc imbalance plays a role in the pathophysiology of IBS and if zinc supplementation may be an effective therapy.

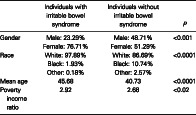

## Introduction

Irritable bowel syndrome (IBS) is common, affecting 12% of the population,^1^ and has a significant economic burden on the health‐care system.^2^ The quality of evidence in support of current medication interventions is rated as very low to moderate.^2^ The majority of individuals with IBS report that their symptoms remain unchanged or worsen over time.^3^ Novel therapies are therefore needed, and increasingly, the initial management has become focused on dietary interventions.

Diet has been assumed to play a role in both the symptomatology and pathophysiology of IBS. Individuals with IBS often attribute symptoms to certain foods,^4^, ^5^ and while the frequency of true food allergies is low, food intolerances are common.^4^, ^6^ One population‐based study found that 70% of those with IBS reported food intolerance, and 62% reported eliminating certain foods from their diet.^4^ It remains unclear whether these dietary avoidances lead to nutritional deficiencies that may in turn contribute to symptoms and associated comorbidities. Prior studies have found that the macronutrient and micronutrient composition of the diet in people with IBS is comparable to those without IBS and far exceeds the recommended dietary intake.^5^, ^7^ However, other studies have found that 12% of individuals with IBS were at risk of adverse health consequences related to their dietary restriction and that they consumed significantly less of certain micronutrients, including zinc, vitamin C, and calcium.^4^, ^5^, ^8^, ^9^ Whether this decreased consumption leads to nutritional deficiencies is unclear. Micronutrient deficiencies, such as vitamin D, have been implicated in the pathology of IBS, and supplementation is being investigated as a potential therapy.^10^ Therefore, understanding the nutritional status of those with IBS and its relation to dietary modifications may identify novel therapeutic targets and suggest novel pathologic pathways.

We aim to compare dietary intake in those with IBS and those without and to evaluate hematologic or biochemical evidence of nutritional deficiencies that may offer opportunities for therapeutic supplementation and potential insight into the pathophysiology of the disease. To do so, we used data from a national survey to examine nutritional intake through a 24‐dietary recall in those with and without IBS and to examine laboratory findings for evidence of malnutrition. The only national survey data we were able to identify with this information was the Second National Health and Nutrition Examination Survey (NHANES II) (1976–1980). Although these data are not current, they provide a unique opportunity for an evaluation of nutritional markers and diet in IBS.

## Methods

There are currently no population‐based studies for IBS that include both dietary recall and hematologic and biochemical studies. Seventeen national health surveys conducted in the United States between 1959 and 2019 were screened to evaluate their suitability in filling this information gap. These surveys were the *Health Examination Surveys* (*n* = 3), the NHANES (*n* = 3) and the *Continuous NHANES* (*n* = 11). Only the NHANES II provided a comprehensive assessment of IBS.^8^ Conducted from 1976 to 1980, this was a nationwide probability sample of participants aged 6 months to 74 years drawn from civilian and noninstitutionalized individuals in the United States and is a multistage sample obtained through a complex sample design that involves clustering and stratification. Adjusted sampling weights allow the results from the analysis of the data to reflect the general noninstitutionalized U.S. population.^11^ Participants underwent a standardized questionnaire and medical exam. and provided blood samples. Invasive procedures such as esophagogastroduodenoscopy and colonoscopy were not performed as part of the survey.

### *Identification of IBS*

Individuals aged 12–74 years were asked if a doctor had ever told them that they had spastic colon or “mucose” colitis. Individuals with IBS were defined as those who responded “yes. Still have it,” “yes. Blank but applicable,” “yes. Don't know if still have it,” and “yes. Does not still have it”. Controls were those who reported that they had never been told that they had spastic colon. Those in either group who reported a history of ulcerative colitis or recurrent/chronic enteritis were excluded. This study focused on adults, and therefore, individuals below the age of 18 were excluded.

### *Poverty income ratio*

The poverty income ratio is the ratio of the total income of the household over the income needed to provide sufficient daily nutritional requirements for that household. The denominator was adjusted for family size, gender of the family head, the age of the family head, year, and the place of residence. Ratios of less than 1.0 were considered below poverty, and ratios greater than or equal to 1.0 were considered at or above poverty. The poverty income ratio was divided into quartiles: 0.01–1.42, 1.42–2.36, 2.36–3.41, and 3.41–8.80.

### *Laboratory values*

This study focused on studies pertinent to nutritional status: serum vitamin B_12_, folate, high‐density lipoprotein (HDL), triglyceride, total cholesterol, copper, zinc, vitamin C, albumin, transferrin, total iron binding capacity (TIBC), iron, mean corpuscular volume (MCV), ferritin, and hemoglobin levels. Both mean values and the odds of having abnormal values were compared between the two groups.

Anemia was defined as a hemoglobin level less than 13 g/dL in men and 12 g/dL in women. Iron deficiency anemia was defined by both anemia and a ferritin level less than 40 ng/mL.

### *Body mass index*

During the physical examination, standing height and body weight were measured. Body mass index (BMI) was calculated as weight in kilograms divided by height in meters squared. Underweight was defined as a BMI less than 18.5, normal weight by a BMI between 18.5 and 24.9, and overweight by a BMI of greater than or equal to 24.9.

### *Reported dietary avoidances*

As part of the medical history questionnaire, individuals were asked if they avoided eating five food products because they disagreed with them: milk, fats/fried foods, green vegetables, seafood, or any other food.

### *Dietary recall*

Two dietary questionnaires were completed with the help of a dietician. The first involved a 24‐h dietary recall, which was used to calculate total calories and the total daily intake of 17 nutrients. The second questionnaire focused on food frequency, looking at weekly consumption rates for various food groups.

The percentage of daily caloric intake dedicated to fat, protein, and carbohydrate was not directly provided in the dataset and was calculated by multiplying the total daily ingested quantity of each macronutrient in grams by 9, 4, and 4 kcal/g respectively and then dividing this by the total daily consumed calories.

### *Statistical analysis*

Multivariate linear regression was performed accounting for the weights, clustering, and strata of the study design. This was adjusted for age (divided into quintiles and treated as a classification variable), gender, race, poverty income ratio, and reported vitamin supplement use by including these variables as covariates. Zinc and copper–zinc ratio analyses were also adjusted for albumin and ferritin levels, with the latter divided into quartiles and treated as a classification variable. Zinc binds to albumin, and a linear relationship has been suggested between the two variables, allowing for albumin‐corrected serum zinc levels.^12^ Albumin was therefore left as a continuous variable during adjustment. Zinc levels are also significantly impacted by inflammation.^13^ The copper–zinc ratio for those with and without IBS was estimated using the least‐squares means method, and a two‐tailed test was used to test for statistical significance in the difference between means. The database used did not have data on C‐reactive protein or erythrocyte sedimentation rate, and therefore, ferritin was used as a marker of inflammation. All statistical analyses were performed through SAS 9.4 (SAS Institute, Cary, NC, USA). *P*‐values less than 0.05 were considered significant.

## Results

A total of 18 447 individuals between the ages of 12 and 74 years were interviewed; 563 reported having been told by a physician that they had IBS. Of these, 304 reported still having symptoms, 226 reported no longer having IBS, 3 answered yes but left the rest of the question blank, and 30 reported yes but were not sure if they still had IBS. Of the individuals interviewed, 17 870 reported never having been told that they had IBS. Individuals with a history of ulcerative colitis or recurrent/chronic enteritis were excluded from both groups, as were those between the ages of 12 and 18, ultimately leaving 413 individuals with a current or past diagnosis of IBS and 11 915 who had no prior diagnosis of IBS.

It can be seen that 48.7% of those without IBS and 23.3% of those with IBS were male (*P* = 0.001). Those with IBS were predominantly European‐Americans compared to those without IBS (97.9% white, 1.9% African‐American, and 0.2% other *vs* 86.7, 10.7, and 2.6% respectively, *P* = 0.0001), and older (45.68 *vs* 40.73 years old, *P* = 0.0001). Individuals with IBS had significantly higher incomes than those without IBS, with a mean poverty income ratio of 2.92 compared to 2.68 (*P* = 0.02) (Table 1). Individuals with IBS were significantly more likely to report supplemental vitamin intake (*P* = 0.003).

**Table 1:** Demographics

	Individuals with irritable bowel syndrome	Individuals without irritable bowel syndrome	*P*
Gender	Male: 23.29%	Male: 48.71%	<0.001
	Female: 76.71%	Female: 51.29%	
Race	White: 97.89%	White: 86.69%	<0.0001
	Black: 1.93%	Black: 10.74%	
	Other: 0.18%	Other: 2.57%	
Mean age	45.68	40.73	<0.0001
Poverty income ratio	2.92	2.68	<0.02

### *Body mass index*

Individuals with and without IBS had a mean BMI of 24.55 and of 25.32 (*P* = 0.014), respectively. Individuals with IBS were more likely to be normal weight than overweight when compared to those without IBS (odds ratio [OR] 0.71, 95% confidence interval [CI] 0.51–0.98) but were not more likely to be underweight (OR 0.73, 95% CI 0.27–1.96).

### *Hematologic and biochemical results*

Individuals with IBS were more likely to have higher copper–zinc ratios (1.70 *vs* 1.55, *P* = 0.048) and were more likely to have copper–zinc ratios greater than 1.8 (OR 1.78, 95% CI 1.02–3.13). While individuals with IBS were more likely to have a lower mean vitamin C level (1.01 *vs* 1.07 mg/dL, *P* < 0.046), they were not more likely to have a vitamin C deficiency (OR 0.29, 95% CI 0.81–2.05).

There was no difference between those with IBS and those without for mean vitamin B_12_ and folate (*P* = 0.82 and 0.40, respectively), and those with IBS were not more likely to have a folate deficiency (OR 0.62, 95% CI 0.28–1.36). There were only three individuals with vitamin B_12_ deficiency, and all of these individuals reported not having IBS. There was no difference in iron, ferritin, TIBC, transferrin saturation, or MCV, and those with IBS were not more likely to have low iron levels, ferritin below 40, macrocytosis, or microcytosis. Serum albumin was not significantly different between the two groups (*P* = 0.32), and individuals with IBS were not more likely to have hypoalbuminemia (OR 2.36, 95% CI 0.29–19.05). Cholesterol panels showed no difference in total cholesterol, HDL, and triglycerides (*P* = 0.75, *P* = 0.92, and *P* = 0.33, respectively) (Tables 2 and 3).

**Table 2:** Mean hematologic and biochemical values for those with and without irritable bowel syndrome (IBS), controlled for age, gender, race, reported vitamin supplementation use, and poverty income ratio

	Normal range^14^	Individuals with IBS	Individuals without IBS	*P*
Hematologic studies				
Hemoglobin (g/dL)	13–17 (men)	14.12	14.11	0.9
	12–15 (women)			
Mean corpuscular volume (fL)	80–100	89.54	89.48	0.85
Iron studies				
Iron (μg/dL)	65–180 (men)	102.07	99.24	0.2
	30–170 (women)			
Ferritin (ng/mL)	12–300 (men)	100.08	110	0.066
	12–150 (women)			
Total iron‐binding capacity (μg/dL)	251.5–474.9	366.15	362.84	0.51
Transferrin saturation (%)	20–50	28.59	27.83	0.28
Gastrointestinal function				
Albumin (g/dL)	3.5–5	4.69	4.67	0.32
Vitamins				
Vitamin B_12_ (ng/L)	130–700	720.16	729.3	0.82
Folate (nmol/L)	7–36	9.04	7.45	0.40
Vitamin C (mg/dL)	0.4–1.5	1.01	1.07	0.046
Lipids				
Total cholesterol (mg/dL)	116–212.68	218.57	217.71	0.75
High‐density lipoprotein (mg/dL)	40–80	51.24	51.17	0.92
Triglycerides (mg/dL)	50–150	143.97	136.26	0.33
Minerals				
Copper (μg/dL)	70–150	127.59	124.87	0.28
Zinc (μg/dL)	66–110	83.16	84.35	0.442
Copper–zinc ratio	0.64–2.27	1.70	1.55	0.048

**Table 3:** Odds ratios and 95% confidence intervals (CI) for abnormal hematologic and biochemical values for those with irritable bowel syndrome compared to those without controlled for age, gender, race, reported vitamin supplementation use, and poverty income ratio

	Odds ratio	95% CI
Anemia (Hgb < 13 for men, Hgb < 21 for women) *versus* normal or elevated Hgb	1.00	0.59–1.71
Microcytosis (MCV < 80) *versus* normal MCV	0.82	0.40–1.68
Macrocytosis (MCV > 100) *versus* normal MCV	0.71	0.39–1.31
Low serum iron (iron < 65 for men, iron < 30 for women) *versus* normal or elevated iron	1.21	0.46–3.22
Hypoferremia (<40) *versus* normal or elevated ferritin	0.73	0.46–1.15
Hypoalbuminemia (<3.5) *versus* normal or elevated albumin	2.36	0.29–19.05
Folate deficiency (<7) *versus* normal or elevated folate	0.62	0.28–1.36
Vitamin C deficiency (<0.4) *versus* normal vitamin C levels	0.29	0.81–2.05
Hypocupremia (<70) *versus* normal copper levels	2.30	0.57–9.33
Hypercupremia (>150) *versus* normal copper levels	0.96	0.64–1.44
Hypozincemia (<66) *versus* normal and high zinc levels†	1.8	0.60–5.39
Copper–zinc ratio > 1.8 compared to levels below 1.8†	1.78	1.02–3.13

† Adjusted for ferritin and albumin levels.

MCV, mean corpuscular volume.

### *Reported dietary avoidances*

Individuals with IBS were significantly more likely to report dietary avoidances. They were more likely to report avoiding milk (OR 1.93, 95% CI 1.34–2.78), fats/fried foods (OR 2.48, 95% CI 1.96–3.14), green vegetables (OR 4.20, 95% CI 2.68–6.59), seafood (OR 2.46, 95% CI 1.31–4.63), and “any other foods” (OR 3.07, 96% CI 2.23–4.21) (Table 4).

**Table 4:** Reported food avoidances in those with irritable bowel syndrome compared to those without, controlled for age, gender, race, and poverty income ratio

	Odds ratio	95% confidence interval
Do you avoid milk because it disagrees with you?	1.93	1.34–2.78
Do you avoid fats/fried foods?	2.48	1.96–3.14
Do you avoid green vegetables?	4.20	2.68–6.59
Do you avoid seafood?	2.46	1.31–4.63
Do you avoid any other foods?	3.07	2.23–4.21

### *Reported food frequency*

Those with IBS consumed significantly less whole milk (*P* = 0.017), skim or butter milk (*P* = 0.02), cheese (*P* = 0.037), ice milk, ice cream, or puddings made with milk (*P* = 0.028) and poultry (*P* = 0.034) (Table 5).

**Table 5:** Reported frequency of intake of food items for those with and without irritable bowel syndrome (IBS), controlled for age, gender, race, and poverty income ratio

	Individuals with IBS	Individuals without IBS	*P*
Mean weekly intake of poultry (times per week)	2.88	3.32	0.034
Mean weekly intake of cheese and cheese dishes (times per week)	3.07	3.55	0.037
Mean weekly intake of ice milk, ice cream, or puddings made with milk (times per week)	3.06	3.56	0.028
Mean weekly intake of skim milk or buttermilk (times per week)	3.04	3.56	0.02
Mean weekly intake of whole milk (times per week)	3.01	3.57	0.017
Mean weekly intake of sugar and primarily sugar products (times per week)	10.97	10.06	0.20
Mean weekly intake of legumes, nuts, and seeds (times per week)	1.36	1.40	0.76
Mean weekly intake of fats and oils (times per week)	11.06	10.16	0.06
Mean weekly intake of organ meats (times per week)	0.37	0.39	0.26
Mean weekly intake of fruits and vegetables (times per week)	1.95	1.84	0.28
Mean weekly intake of fish and shellfish (times per week)	1.18	1.20	0.74
Mean weekly intake of meat (times per week)	5.38	5.38	0.99
Mean weekly intake of grain products (times per week)	12.34	12.30	0.95
Mean weekly intake of cereals (times per week)	2.15	2.07	0.59

### *Reported dietary intake*

Individuals with IBS reported a total daily caloric intake of 1891.45 kilocalorie, while those without reported 1794.59 kilocalorie (*P* = 0.022). Those with IBS reported that 16.6% of their daily caloric intake came from protein, 47.2% from carbohydrates, and 36.2% from fat, while those without reported 17, 46.9, and 36.1%, respectively (*P* = 0.33, *P* = 0.72, *P* = 0.8, respectively) (Table 6).

**Table 6:** Mean total reported daily calories, adjusted for age, gender, race, and poverty income ratio

	Individuals with irritable bowel syndrome	Individuals without irritable bowel syndrome	*P*
Total reported daily calories	1891.45 kcal	1794.59 kcal	0.022
Percentage of diet from protein	16.6%	17%	0.33
Percentage of diet from carbohydrates	47.2%	46.9%	0.72
Percentage of diet from fat	36.2%	36.1%	0.8

Compared to individuals without IBS, those with IBS had similar intakes of all the analyzed micro‐ and macronutrients. They consumed similar cholesterol, linoleic acid, oleic acid, and saturated fatty acid (*P* = 0.291, *P* = 0.055, *P* = 0.15, and *P* = 0.41, respectively). They also had no significant difference in vitamin intake, specifically vitamin C, niacin, riboflavin, thiamine, and vitamin A (*P* = 0.86, *P* = 0.64, *P* = 0.46, *P* = 0.55, and *P* = 0.53, respectively) (Table 7). The consumption of the minerals potassium, sodium, iron, phosphorus, and calcium was also not significantly different between the two groups (*P* = 0.86, *P* = 0.31, *P* = 0.53, *P* = 0.57, and *P* = 0.51, respectively) (Table 7).

**Table 7:** Reported mean daily intake of micro‐ and macronutrients for those with and without irritable bowel syndrome (IBS), controlled for age, gender, race, and poverty income ratio

	Individuals with IBS	Individuals without IBS	*P*
Daily ingested cholesterol (mg)	367.12	352.43	0.291
Daily ingested linoleic acid (g)	10.72	10.05	0.055
Daily ingested oleic acid (g)	29.01	27.83	0.15
Daily ingested saturated fatty acid (g)	26.94	26.23	0.41
Daily ingested vitamin C (mg)	110.17	108.94	0.86
Daily ingested niacin (mg)	19.58	19.24	0.64
Daily ingested riboflavin (mg)	1.68	1.64	0.46
Daily ingested thiamine (mg)	1.28	1.25	0.55
Daily ingested vitamin A (international units)	6059.82	5843.5	0.53
Daily ingested potassium (mg)	2247.81	2234.98	0.86
Daily ingested sodium (mg)	2862.6	2757.22	0.31
Daily ingested iron (mg)	12.92	12.62	0.53
Daily ingested phosphorus (mg)	1147.54	1130.73	0.57
Daily ingested calcium (mg)	655.04	637.91	0.51

## Discussion

This study aimed to determine if dietary avoidances place those with IBS at risk of nutritional deficiencies and if these deficiencies suggest a need for therapeutic supplementation. While individuals with IBS reported significantly more dietary avoidances, analysis of their dietary recall indicated that their consumption of micro‐ and macronutrients was not significantly different than those without IBS, and in fact, they consumed significantly more daily calories. While mean BMI was lower in those with IBS, it was still within normal range, and individuals with IBS were not more likely to be underweight. Individuals with IBS were significantly more likely to have an elevated copper–zinc ratio, reflective of underlying copper–zinc imbalance. Only one other study could be identified that examined the copper–zinc ratio in individuals with IBS. Although this study found significantly less zinc intake in those with IBS, they found no difference in the copper–zinc ratio. However, this result may have been confounded as the study population was restricted to adolescent girls, and the control group included girls with premenstrual syndrome or primary dysmenorrhea, as well as “healthy” controls.^8^ Another study identified zinc deficiency in the subgroups of those with IBS.^15^

The higher total energy intake in individuals with IBS and similar BMI and macro‐ and micronutrient intake have been documented in several other studies.^5^, ^7^, ^9^, ^16^ This remains controversial, however, as others have identified increased fat intake in those with IBS and decreased micronutrient intake of calcium, zinc, iron, potassium, phosphorus, vitamin C, and several B vitamins.^9^, ^17^ Notably, hematologic and biochemical analysis in our study did not identify differences in serum calcium, iron, potassium, or phosphorus levels between those with and without IBS. While vitamin C levels were lower in those with IBS, vitamin C deficiency was not more likely in this population.

Avoidance of dairy products among IBS individuals may explain the high copper–zinc ratio identified in this study. Meat and milk (both skim and whole) were the most significant sources of dietary zinc in the studied population, accounting for 56–60% of total intake.^18^ While the weekly consumption of meat was not different between those with and without IBS, those with IBS consumed significantly less milk. Consumption of other food items high in zinc, such as fish and shellfish, and consumption of foods that can inhibit zinc absorption due to the presence of phytates, such as whole grains and cereals, were not different between the two groups. Several other studies have similarly found that individuals with IBS consumed significantly fewer milk products and that this in turn led to less calcium and, in one 2018 study, less zinc intake.^9^, ^17^, ^19^ Milk remains one of the most common food triggers and most commonly avoided food item for those with IBS.^4^, ^19^ While other studies have identified avoidance of foods such as yogurts, fruits, and eggs, these food groups were either not studied in our database or were grouped with other foods.

While at this point hypothetical, the significantly elevated copper–zinc ratios have potential causal implications for the associated psychiatric conditions and pathophysiology of IBS that should be further investigated. Both zinc and copper have important roles in human physiology, specifically in enzyme function, and the copper–zinc ratio has more clinical implications than the actual concentration of either mineral.^20^, ^21^ One enzyme in particular, copper/zinc superoxide dismutase 1, relies on the copper and zinc ratio to function properly, and imbalances of these minerals predisposes to a chronic inflammatory state, which is one proposed pathophysiologic factor in IBS. An elevated copper–zinc ratio has been identified as a marker of increased oxidative stress and inflammation.^21^ Elevated copper–zinc ratios suggest an imbalance and, more specifically, a deficiency of circulating zinc relative to copper levels. In addition, an elevated ratio has also been demonstrated in the setting of zinc deficiency.^22^ We did not find a difference in plasma zinc between those with IBS and those without; however, levels of zinc in the blood have poor sensitivity and specificity for zinc deficiency, and marginal deficiency can be difficult to identify. In addition, clinical evidence of zinc deficiency can be present despite normal serum levels.^20^ It is unclear if the elevated copper–zinc levels identified in those with IBS were due to a relative imbalance of circulating copper and zinc or due to a marginal zinc deficiency that was not picked up in the biochemical studies. Unfortunately, more sensitive tests for zinc deficiency were not collected as part of the NHANES II survey.

Abnormal copper–zinc ratios have been identified in a number of the psychiatric conditions that overlap with IBS, including chronic fatigue, depression, and anxiety,^23^, ^24^ and zinc deficiency has been identified as the possible causal factor for the “brain–gut” axis in autism spectrum disorder.^25^ Copper and zinc are neurotransmitters, and their ratio has been correlated with symptom severity in anxiety and autism spectrum disorders.^23^, ^24^, ^26^, ^27^, ^28^ Similar to IBS, there appears to be a “brain–gut” interaction in autism spectrum disorders, with an estimated 42% having gastrointestinal complaints^25^ and with the severity of these symptoms being associated with the severity of autistic symptoms. Zinc deficiency has been proposed as an explanation for this “brain–gut” axis due to the postulated role of high copper and low zinc in modulating the *gamma*‐Aminobutyric acid (GABA) system,^25^ leading to a lower transmitter concentration and hyperactivity.^27^, ^29^ The hypothesized role of GABA in visceral pain, gastrointestinal motility, and visceral sensation^30^ provides a hypothetical explanation for the role of zinc in the “brain–gut” axis of IBS. Zinc supplementation may be of therapeutic benefit in IBS as it has been found to normalize the copper–zinc ratio and improve symptoms in anxiety and in autism spectrum disorders with concurrent gastrointestinal symptoms.^24^, ^29^

The copper and zinc imbalance and relative zinc deficiency identified in this study may modulate or perpetuate the pathophysiology of IBS, specifically through its role in increased small‐bowel mucosal permeability,^31^ altered microbiome, and carbohydrate malabsorption, which are postulated etiological mechanisms in IBS. Zinc is crucial to mucosal integrity through its actions on epithelial tight junctions and barrier function,^32^ and zinc supplementation has been found to improve mucosal integrity in several conditions affecting the small intestine.^33^, ^34^ Zinc deficiency and supplementation have been found to have significant impacts on the gut microbiome.^35^ In addition, chronic zinc deficiency has been shown to reduce the activity of disaccharidases by 30–50%. Proper function of these enzymes is needed for the digestion of carbohydrates and absorption of saccharides. Zinc supplementation may therefore provide an opportunity to mitigate the underlying pathology of IBS and improve symptoms.

This study has several limitations. One potential limitation is that individuals with IBS were identified through self‐reporting and not by symptom‐based criteria or through evaluation by a physician. Self‐reporting is at risk of recall bias and is influenced by the medical literacy, health awareness, and health‐seeking behavior of individuals. In addition, the diagnostic criteria for IBS at the time of the survey are different from our current criteria. While the Manning criteria, published in 1978, differ from our current Rome III criteria, published in 2006, one study found that 90% of cases of IBS identified through Rome III were also identified by the Manning criteria, suggesting significant agreement between the two methods.^36^ While it is possible that the individuals with IBS were diagnosed using the Manning criteria, this cannot be verified.

Another potential limitation is that the data used for this study were collected in 1976–1980; however, this was the last NHANES dataset to identify individuals with IBS and the only one to also include serum copper and zinc measurements. In addition, we could not exclude celiac disease, which has an estimated prevalence of 5.7% among individuals with IBS^37^ compared to 1% in the general population. Those with celiac disease are at increased risk of copper deficiency and have been found to have normal zinc levels,^38^, ^39^ so the inability to exclude them from the group with IBS may have biased our estimates of the copper–zinc ratio in the group with IBS toward zero. Another potential limitation is recall bias, which may have affected the results of the 24‐h dietary recall and food frequency survey. However, our finding of an abnormal copper–zinc ratio was based on hematologic and biochemical testing, which would not be affected by this bias.

In conclusion, individuals with IBS reported more dietary intolerances and consumed significantly less poultry and milk. They were significantly more likely to have a high copper–zinc ratio, reflective of a zinc deficiency relative to circulating copper levels. While zinc has been previously suggested as a therapeutic option in IBS, no studies have been performed to examine its effectiveness.^40^ We hope that our findings motivate further studies to investigate whether identifying and treating the abnormal copper–zinc ratio may improve associated symptoms and modulate associated comorbid psychiatric conditions, as well as the underlying pathophysiology of IBS.
